# Emerging concepts in liquid biopsies

**DOI:** 10.1186/s12916-017-0840-6

**Published:** 2017-04-06

**Authors:** Samantha Perakis, Michael R. Speicher

**Affiliations:** 1 Institute of Human Genetics, Medical University of Graz, Harrachgasse 21/8, A-8010 Graz, Austria; 2 BioTechMed, Graz, Austria

**Keywords:** Cell-free DNA, Circulating tumor DNA, Circulating tumor cells, Liquid biopsy, Disease monitoring, Precision medicine

## Abstract

Characterizing and monitoring tumor genomes with blood samples could achieve significant improvements in precision medicine. As tumors shed parts of themselves into the circulation, analyses of circulating tumor cells, circulating tumor DNA, and tumor-derived exosomes, often referred to as “liquid biopsies”, may enable tumor genome characterization by minimally invasive means. Indeed, multiple studies have described how molecular information about parent tumors can be extracted from these components. Here, we briefly summarize current technologies and then elaborate on emerging novel concepts that may further propel the field. We address normal and detectable mutation levels in the context of our current knowledge regarding the gradual accumulation of mutations during aging and in light of technological limitations. Finally, we discuss whether liquid biopsies are ready to be used in routine clinical practice.

## Background

As the concept of precision medicine in the field of cancer management continues to evolve, so too do the challenges and demands with regards to diagnosis, prognosis, and prediction of treatment resistance [1, 2]. Although the discovery of molecular agents able to target specific genomic changes in metastatic cancer patients has revolutionized patient care, tumor heterogeneity remains a daunting obstacle for clinicians who need to optimize therapy regimens based on an individual’s cancer genome [3]. Tissue biopsies, which still currently represent the standard of tumor diagnosis, unfortunately only reflect a single point in time of a single site of the tumor. Such a sampling method is thus inadequate for the comprehensive characterization of a patient’s tumor, as it has been demonstrated that various areas within the primary tumor or metastases can in fact harbor different genomic profiles [4]. The molecular genetic diversity within a tumor can also alter over time, thus rendering future treatment decisions based on historical biopsy information potentially inaccurate and suboptimal [5, 6]. Furthermore, a surgical biopsy procedure is hampered by limited repeatability, patient age and comorbidity, costs, and time, potentially leading to clinical complications. Despite these ongoing clinical issues, the advent of next-generation sequencing (NGS) technologies has proven its value in the search for novel, more comprehensive and less invasive biomarkers in order to truly realize the goals of cancer precision medicine [1].

Such minimally invasive tests, known as a “liquid biopsies” [7, 8], have gained plenty of traction in the last few years and the method was even recently listed as a top ten technology breakthrough in 2015 by the MIT Technology Review (www.technologyreview.com/s/544996/10-breakthrough-technologies-of-2015-where-are-they-now/). One strategy of this approach takes advantage of circulating free DNA (cfDNA) found in the plasma component of blood to evaluate the current status of the cancer genome. Since the discovery of the existence of cfDNA in 1948, numerous research efforts have attempted to harness this easily accessible and rich genetic information in the circulation of cancer patients. Furthermore, other components, such as circulating tumor cells (CTCs) or exosomes, have been intensively investigated. Herein, we briefly summarize the current technologies and applications, the detection rates in the context of the number of mutations that is normal for healthy individuals depending on their age, and the new technologies and emerging concepts as well as existing challenges for liquid biopsy applications. Finally, we will present our view as to when the information from liquid biopsies will be reliable and clinically applicable.

## Current technologies and applications

Here, we refer to technologies as “current” if they can be viewed as established approaches reflected in several publications describing their applicability. In contrast, “emerging technologies” are novel ideas and concepts for which proof-of-concepts or only a few applications have been published. Current technologies applied in liquid biopsy research have been extensively reviewed [9–12] and we have therefore only briefly summarized them herein.

### Circulating tumor DNA (ctDNA)

Technologies based on the analysis of ctDNA can be mainly classified as targeted or untargeted (Table 1). Targeted approaches are used to analyze single nucleotide mutations or structural chromosomal rearrangements in specified genomic regions of plasma DNA and to estimate the allelic frequency of a particular mutation within a sample. For example, somatic mutation profiling can be performed by quantitative or digital PCR. Using digital PCR, ctDNA could be detected in > 75% of patients with advanced cancers and in 48–73% of patients with localized tumors [13]. Although digital PCR-based methods have demonstrated to have suitable clinical sensitivity considering that digital PCR and BEAMing (beads, emulsion, amplification, and magnetics) can detect somatic point mutations at a sensitivity range of 1% to 0.001% [14], these technologies require prior knowledge of the region of interest to detect known mutations given the need for the PCR assay to be designed accordingly. Furthermore, digital PCR is limited by scalability for larger studies. In particular, chromosomal rearrangements have demonstrated an excellent sensitivity and specificity [15, 16]. The PARE (personalized analysis of rearranged ends) approach first requires the identification of specific somatic rearrangements, i.e., breakpoints, found in the tumor followed by the development of a PCR-based assay for the detection of these events in cfDNA [15]. As these genomic rearrangements are not present in normal human plasma or tissues unrelated to the tumor, their detection has a high specificity and sensitivity. A downside of this approach is that such rearranged sequences must not be driver events and may get lost during a disease course and therefore may not reflect the evolution of the tumor genome [15, 16].

**Table 1:** Summary of some current technologies, their main applications, and some representative references

Approach	Purpose	Reference
Targeted ctDNA approaches		
Digital PCR, BEAMing (beads, emulsion, amplification, and magnetics)	Detection of specific mutations	[13, 14]
PARE (personalized analysis of rearranged ends)	Detection of specific structural chromosomal rearrangements	[15, 16]
Gene panels, TAm-Seq (tagged amplicon deep sequencing), CAPP-Seq (cancer personalized profiling by deep sequencing), Safe-SeqS (Safe-Sequencing System)	Detection of mutations in a predefined gene panel	[17, 19, 22, 29, 60, 97]
Untargeted ctDNA approaches		
Whole-exome sequencing	Analysis of all protein coding genes; copy number alterations	[20]
Whole-genome sequencing	Copy number alterations; dependent on sequence depth identification of mutations	[21, 22, 24]
Circulating tumor cells		
Whole-exome sequencing	Analysis of all protein coding genes; copy number alterations	[105, 106]
Whole-genome sequencing; array-CGH	Copy number alterations	[26, 34, 88, 106]
In situ RNA-FISH	Detection of specific transcripts	[37]
qRT-PCR	Evaluation of specific transcripts	[40]
Microfluidics, Arrays	Transcriptional profiling	[35, 37, 38]
Exosomes		
DNA	Tumor genome profiling	[45, 49]
RNA	Transcription profiling	[45, 49]
Protein	Protein marker analysis	[50]

Therefore, several NGS-based strategies have been developed not for targeting single or a few specific mutations, but rather for selected, predefined regions of the genome by employing gene panels. In principle, any gene panel can be applied to cfDNA; however, in order to increase resolution for mutations occurring with low allele frequency, special technologies have been developed. TAm-Seq (tagged amplicon deep sequencing) amplifies entire genes by tiling short amplicons using a two-step amplification and produces libraries tagged with sample-specific barcodes [17]. Through this method, the detection of cancer-specific mutations down to allele frequencies as low as 2% and of known hotspot mutations in *EGFR* and *TP53* down to approximately 0.2% has been reported [17, 18]. The CAPP-Seq (cancer personalized profiling by deep sequencing) method was applied to patients with non-small cell lung cancer (NSCLC) and detected ctDNA in 100% of stage II–IV NSCLC patients as well as in 50% of stage I patients [19].

In contrast, untargeted approaches do not depend on a priori knowledge and aim at a comprehensive analysis of the tumor genome. One approach involves whole-exome sequencing, which can be adopted for sequencing of cfDNA for the identification of clinically actionable mutations [20]. Whole-genome sequencing of plasma DNA allows the comprehensive characterization of structural variations and somatic copy number alterations (SCNAs) [21–24]. These assays have similarities to “digital karyotyping”, which involves digital enumeration of observed so-called “tag sequences” from specific genomic loci along each chromosome [25]. Such a read-depth analysis by “tag-counting” has been the underlying principle for the implementation of whole-genome sequencing approaches using plasma DNA to identify copy number changes associated with tumor genomes [21, 22, 24, 26–29]. Interestingly, for SCNA applications, a shallow sequencing depth of approximately 0.1–0.2× is sufficient for analyses [22].

### Circulating tumor cells (CTCs)

A second approach to liquid biopsy research examines whole tumor cells in the bloodstream, known as CTCs [30, 31]. The first account of the existence of CTCs in the blood came from Thomas Ashworth in 1869, much earlier than the first mentioning of cfDNA, in which he postulated that these cells might potentially shed light onto the mystery behind metastases in an individual with cancer. Although a multitude of devices for isolation of CTCs have been described [30, 32], only the CellSearch system (Janssen Diagnostics) has been approved by the FDA to date. Previously, it was thought that enumeration of tumor cells in the blood could be used alone as a barometer to measure the level of aggressiveness of a particular cancer; however, improvement of NGS and isolation methodologies has allowed analyses of DNA and RNA from isolated cells in order to gain insight into cancer driver genes (Table 1). Since single-CTC analyses have provided evidence of genetic heterogeneity at the level of an individual cell, many studies have investigated their diagnostic potential and application in cancer management [33–39].

A strength of CTC analyses is that, as a single-cell approach, not only pure tumor DNA but also pure tumor RNA can be obtained. This greatly facilitates the analyses of splice variants, which, for example, play an important role in the development of resistance to androgen deprivation therapies in men with prostate cancer [35, 40].

### Exosomes

A third target of liquid biopsies involves exosomes, which are circulating vesicles harboring nucleic acids shed by living cells as well as tumors. Exosomes can range from 30 to 200 nm in size and can be isolated from plasma, saliva, urine, and cerebrospinal fluid as well as from serum [41, 42]. The exosome field has gained recent attention since several studies have demonstrated that these actively released vesicles can function as intercellular messengers [43–46]. Since they are stable carriers of DNA, RNA, and protein from the cell of origin (Table 1), this makes them particularly attractive as biomarkers of cancer. Tumor exosomes, in particular, have been linked to the stimulation of tumor cell growth, immune response suppression, and induction of angiogenesis [43], and have been shown to play a role in metastasis [47, 48]. Because tumor cells actively shed tens of thousands of vesicles a day, it has been estimated that hundreds of billions of vesicles can be found in a milliliter of plasma [45]. Moreover, exosomes can harbor RNA with tumor-specific mutations [43, 45, 49] and DNA originating from these vesicles can be used to detect both gene amplifications and mutations [45, 49].

Importantly, exosomes may have the potential to detect very early cancer stages, as recently shown in patients with pancreatic cancer [50]. Using mass spectrometry analyses, glypican-1 (GPC1) was identified as a cell surface proteoglycan, which was specifically enriched on cancer-cell-derived exosomes. GPC1^+^ circulating exosomes carried specific *KRAS* mutations distinguishing healthy subjects and patients with a benign pancreatic disease from patients with early- and late-stage pancreatic cancer. Furthermore, these exosomes allowed the reliable detection of pancreatic intraepithelial lesions at very early stages despite negative signals by magnetic resonance imaging, which may enable curative surgical interventions in this otherwise dismal disease [50].

## Mutation baseline value in healthy individuals

A great promise attributed to liquid biopsies is their possible potential to detect cancer early or even to detect precursor lesions before clinical signs occur or before sophisticated imaging systems are able to detect them. However, a major problem is the number of somatic mutations that occur in healthy individuals.

The question regarding what constitutes typical somatic variation and to what extent does it take form in terms of phenotype has gained attention by recent landmark large-scale studies [51, 52]. Interestingly, it is possible for healthy individuals to harbor disadvantageous variants without exhibiting any apparent disease phenotype [51, 52]. In fact, the identification of rare homozygotes that predicted loss of function genotypes revealed that loss of most proteins is relatively harmless to the individual [52]. The Exome Aggregation Consortium study analyzed high-quality exome sequencing data from 60,706 individuals of diverse geographic ancestry and identified 3230 genes highly intolerant to loss-of-function. Interestingly, 72% of these genes have no established human disease phenotype yet [51]. Thus, despite our growing knowledge about the human genome, identified variants require cautious interpretation with respect to the potential consequences for the phenotype.

In the context of cancer and according to the somatic mutation theory of cancer [53], malignant diseases are, to a large extent, the result of acquired genetic and epigenetic changes, which has now been extensively confirmed by NGS technologies [54, 55]. However, a tremendous challenge is the measurement of the somatic mutation rate in normal tissue and establishment of baseline values, i.e., what number of mutations is normal for a healthy person at a certain age. In general, somatic mutation rates are higher than germline mutation rates. For example, it is estimated that, in humans, the per generation rate in intestinal epithelium or fibroblasts/lymphocytes is approximately 13- and 5-fold, respectively, greater than in the germline [56].

As somatic mutations occur in individual cells, each mutation represents a low frequency event and special NGS methods for detection of such rare mutations are needed. Promising approaches include single cell genomic sequencing [6, 34, 57–59] and applications of molecular barcodes [60, 61]. The bottleneck sequencing system is a novel technology that allows the quantification of somatic mutational load in normal human tissues, even at a genome-wide level [62]. The bottleneck is created by dilution of a sequencing library before PCR amplification, resulting in random sampling of double-stranded template molecules. This increases the signal of a rare mutation compared to wild-type sequences and thus enables the detection of mutations occurring at 6 × 10^–8^ per base pair. With this approach, it was shown that, in normal colonic epithelium, mutation rates in individuals over 91 years of age had increased by an average of 30-fold in mitochondrial DNA and 6.1-fold in nuclear DNA [62]. Importantly, the spectra of rare mutations in normal colon and kidney tissues were similar to those of the corresponding cancer type [62], confirming previous reports that cancer-associated mutations may also occur in normal stem cells [63, 64].

Thus, direct measurements of mutations in adult stem cells are necessary, as the gradual accumulation of mutations in adult stem cells is thought to have an especially large impact on the mutational load of tissues due to their potential for self-renewal and capacity to propagate mutations to their daughter cells [63]. Indeed, statistical analyses have recently suggested that the total number of divisions of adult cells needed to maintain tissue homeostasis correlates with the lifetime risk of cancer [63]. However, these calculations could not exclude extrinsic risk factors as additional important determinants for cancer risk [65].

**Fig. 1:**
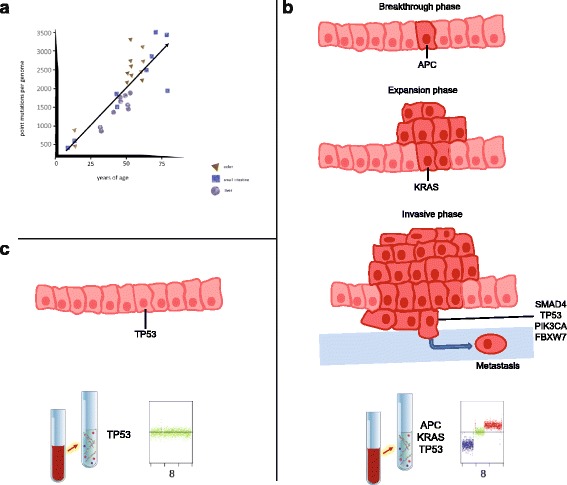
Mutation rate in adult stem cells and their potential consequences. **a** Correlation of the number of somatic point mutations in adult stem cells derived from colon, small intestine, and liver with age of the donor (adapted from [66]); there is an increase of ~36 mutations/adult stem cell/year. **b** Summary of the “Three strikes to cancer model” [68] for colorectal cancer, where mutations occur in specific driver genes. In the breakthrough phase, a mutation occurs in *APC* and results in abnormal division of the respective cell. Subsequently, a mutation in *KRAS* may follow in the expansion phase and may give rise to a benign tumor. Occurrence of a further mutation in a driver gene in at least one of the listed pathways *SMAD4*, *TP53*, *PIK3CA*, or *FBXW7* may enable the tumor to invade surrounding tissues and to initiate the invasive phase with dissemination of tumor cells and formation of metastases [68]. The mutations may be detectable in cfDNA; furthermore, depending on the ctDNA allele frequency and tumor stage, somatic copy number alterations may become visible (shown exemplarily for chromosome 8: blue: lost; green: balanced; and red: gained region). **c** As the order of driver gene mutations is important, the consequences differ if a *TP53* mutation occurs in a colon stem cell before the initiating mutations have taken place. Such a *TP53* mutation alone will not be sufficient to cause increased proliferation or even to transform the cell into a tumor cell. However, due to the stem cell’s capacity of self-renewal, cells with this mutation may be propagated in the respective part of the colon. Depending on how many of these cells are removed by apoptosis or other events, ultra-sensitive ctDNA assays may then detect this mutation in the blood; this will usually not be accompanied by copy number alterations (as indicated by the green scatter-plot for chromosome 8)

Measurement of the somatic mutation load in stem cells within various human tissues poses an immense technical problem. Blokzijl et al. [66] addressed this challenge by using cells capable of forming long-term organoid cultures. An organoid can be defined as a cellular structure containing several cell types that have developed from stem cells or organ progenitors that self-organize through cell sorting and spatially restricted lineage commitment [67]. Single adult stem cells from the small intestine, colon, and liver, tissues which differ greatly in proliferation rate and cancer risk, were expanded into epithelial organoids to obtain sufficient DNA for whole-genome sequencing. The donors ranged in age from 3 to 87 years and, not unexpectedly, it was found that stem cells accumulated mutations with age independent of tissue type [66]. The mutation rate, i.e., the increase in the number of somatic point mutations in each stem cell, was in the same range for all assessed tissues, at approximately 36 mutations per year, despite the large variation in cancer incidence among these tissues (Fig. 1a). Importantly, the findings suggested a universal genomic ageing mechanism, i.e., a chemical process acting on DNA molecules, independent of cellular function or proliferation rate. Furthermore, this intrinsic, unavoidable mutational process can cause the same types of mutations as those observed in cancer driver genes [66].

Given the high mutation rate in adult stem cells, it may be surprising that cancer incidence is not actually higher. According to the “Three Strikes and You’re Out” theory [68] (Fig. 1b), alterations in as few as three driver genes may be sufficient for a cell to evolve into an advanced cancer. However, several reasons may account for the relatively low cancer incidence. First, mutations in stem cells are non-randomly distributed and associated with depletion in exonic regions. Second, if a mutation occurs in an exonic region, it has to be in a cancer driver gene and only a small number of genes in the human genome have been shown to act as driver genes [69]. Third, the order in which driver gene mutations accumulate is important, meaning that mutations which are initiating events have to occur first [68]. Fourth, many of the initiating driver gene mutations are tissue specific; thus, the driver gene mutation must occur in the right gene and not in any driver gene.

In light of these findings, it is not surprising that cancer-associated mutations might be identified in plasma DNA from healthy persons. This was shown in a recent study that used an assay specifically designed to accurately detect *TP53* mutations at very low allelic fractions, in which cfDNA *TP53*-mutated fragments were found in 11.4% of 123 matched non-cancer controls [70] (Fig. 1c). However, the detection of low-allele variants may be impeded by background errors occurring during library preparation and/or sequencing. To address this, approaches such as molecular barcoding and background reduction by sophisticated bioinformatics methods have been developed, as is discussed below.

## New liquid biopsy technologies and emerging concepts

### Improved low-frequency allele detection

One of the biggest technical challenges to overcome in the analysis of cfDNA is the issue of low-frequency mutant alleles since ctDNA levels vary greatly among patients and can reach as low as 0.01% of the total cfDNA in patients with early-stage disease [7, 10]. Although massively parallel sequencing technologies in principle offer the capacity of detecting these singled out rare variants, the error rate of sequencing instruments is typically a limiting factor for accurately calling these variants. Therefore, the application of molecular barcodes has received much warranted attention in the last few years [17, 19, 60, 61] and the resolution may be further increased by bioinformatics approaches.

For example, Newman et al. [71] expanded on their existing CAPP-Seq method by adding a molecular barcode approach and by incorporating an in silico bioinformatics strategy to reduce background noise, which they dubbed “integrated digital error suppression”. They were able to increase the sensitivity of the original CAPP-Seq method by 15-fold and reported a sensitivity and specificity of 92% and 96%, respectively, when profiling *EGFR* kinase domain mutations in cfDNA of NSCLC samples. However, it must be considered that a typical plasma sample of 1 mL contains approximately 3000 copies of each gene, implicating a sensitivity limit of detecting only 1 in 15,000 copies from a 5-mL sample [72]. Including statistical sampling errors, the available genome equivalents of clinical samples will be an important determinant of possible resolution limits in ctDNA analyses.

Nevertheless, novel commercial products, including molecular barcodes, are being offered by industry providers (e.g., ThruPLEX® Tag-seq, Rubicon Genomics; HaloPlex^HS^, Agilent; QIAseq Targeted DNA Panels, Qiagen) and may help to make these sophisticated technologies broadly available. Another large-scale initiative known as GRAIL (www.grailbio.com) vows to detect cancer so early that it can be cured. This ambitious aim is supposed to be accomplished by efforts including ultra-broad and ultra-deep sequencing, bioinformatics, and large population-based clinical studies [73].

### Epigenetics: plasma bisulfite sequencing and nucleosome mapping

**Fig. 2:**
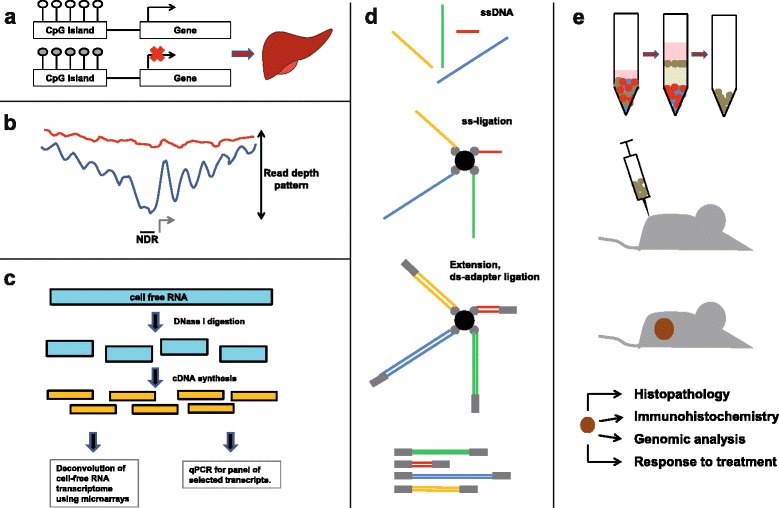
Summary of some emerging technologies in the liquid biopsy field. **a** Plasma DNA tissue mapping: Plasma DNA tissue mapping is an approach employing genome-wide bisulfite sequencing of plasma DNA and methylation deconvolution of the sequencing data to trace the tissue of origin of plasma DNA in a genome-wide manner (here shown exemplarily for liver-specific markers). The signal-to-noise ratio of such assays can be increased by the analysis of stretches of several CpG sites adjacent to the tissue-specific methylation marker. **b** Nucleosome mapping: analysis of the genomic sequencing coverage of plasma DNA fragments reveals the position of nucleosomes because plasma DNA is nucleosome-protected DNA. At transcription start sites (TSSs; indicated by a gray arrow), in particular at the nucleosome depleted region, the read depth is lower and has distinct coverage patterns around the TSSs of highly expressed genes (shown in blue), which differs from coverage patterns of unexpressed genes (red). **c** Plasma RNA-seq: After extraction of cell-free RNA from plasma and DNase I digestion, cDNA is synthesized and amplified from cell-free RNA. Deconvolution of the cell-free transcriptome using microarrays is conducted to determine the relative RNA contributions of certain tissues in a sample based on known tissue-specific expression profiles. In parallel, selected transcripts are quantified by qPCR (procedure based on [81]). **d** Single-stranded DNA (ssDNA) library preparation: the scheme illustrates the key steps in the ssDNA ligation procedure. The ssDNA (top panel), which is not size-selected to avoid elimination of shorter fragments, is ligated to biotinylated probes (second panel), and after ligation of double stranded primers, extended to double-stranded DNA (third panel). DNA molecules of different lengths with a lower limit of efficient capture of approximately 40–60 bp can be obtained (adapted from [86]). **e** CTC-derived explants (CDXs): The patients’ blood is enriched for CTCs (green cells in top panel) and injected into one or both flanks in mice (second panel). The obtained CDXs (brown tumor in third panel) are then analyzed by histopathology, immunohistochemistry, and genomic analyses to confirm that characteristics of the original tumor were maintained. Mice bearing CDXs can be treated to evaluate response to various agents

Of especial interest are studies of cfDNA methylation patterns, since plasma contains a mixture of DNA from different tissues and organs. As certain methylation patterns are tissue specific, they could serve as an epigenetic signature for the respective cells or tissues that release their DNA into the circulation. Such efforts greatly benefit from reference methylomes of multiple tissue types provided by the International Human Epigenome Consortium. For example, “plasma DNA tissue mapping” is an approach employing genome-wide bisulfite sequencing of plasma DNA and methylation deconvolution of the sequencing data to trace the tissue of origin of plasma DNA in a genome-wide manner [74]. In order to increase the signal-to-noise ratio of such assays, stretches of four to nine CpG sites adjacent to the tissue-specific methylation marker site can be used [75] (Fig. 2a). Indeed, such a procedure may achieve sensitivities suitable not only for cancer detection but also for other clinical conditions such as type I diabetes, multiple sclerosis, acute brain damage following cardiac arrest, or traumatic brain injury [75].

A recent study took a very different approach to whole-genome sequencing and leveraged the fact that plasma DNA is nucleosome-protected DNA. This is reflected in the genomic sequencing coverage of plasma DNA fragments around transcription start sites (TSSs), as read depth was lower and had distinct coverage patterns around the TSSs of housekeeping genes and other highly expressed genes. The sequencing coverage differed from unexpressed genes, which are densely packed by nucleosomes [76] (Fig. 2b). In fact, nucleosome positions inferred from whole-genome sequencing of plasma DNA strongly correlated with plasma RNA levels in cancer-free subjects. Furthermore, in plasma of patients with cancer the expression levels of genes in the corresponding tumor were reflected by the coverage around the TSSs [76].

In addition, Snyder et al. [77] also recently identified a direct association between cfDNA and nucleosome positioning and similarly demonstrated that cfDNA levels and fragment sizes reflected the epigenetic features characteristic of lymphoid and myeloid cells. These current studies both expand on the potential of using ctDNA analysis for other applications rather than just mutation or SCNA analysis. New possibilities arise from these findings such as the investigation of a patient’s individual cancer transcriptome, tracking changes in gene isoform expression during treatment, or even helping identify the tissue of origin in cancers which the primary tumor is unknown [78].

### Plasma RNA analyses

Plasma cell-free RNA has been investigated for a long time [79, 80]; however, the comprehensive RNA analyses to establish landscapes of cell-free RNA transcriptomes either by microarrays or by RNA sequencing (RNA-seq) is relatively novel (Fig. 2c). These technologies are promising as they can provide insights into the temporal dynamics of plasma mRNA and, furthermore, analyses of tissue-specific genes allows for estimation of the relative contributions of tissues that contribute circulating RNA. This may enable monitoring of some developmental or disease states of certain tissues; for example, cell-free RNA patterns were longitudinally analyzed in pregnant women and after delivery [81, 82]. However, RNA transcription can vary between people with different variables such as sex, age, or certain diseases. Therefore, carefully annotated health control libraries from individuals with various health conditions are needed for comparison of diseases such as cancer [83].

### Novel plasma DNA preparation protocols

In most protocols, cfDNA is adapted for sequencing via ligation of double-stranded DNA adapters. However, recent studies have provided evidence that ctDNA is shorter than cfDNA from non-tumor cells [84, 85]. As double-stranded DNA library preparations are relatively insensitive to ultrashort, degraded cfDNA, it has been suggested that single-stranded DNA library preparation may represent an alternative and may yield increased proportions of smaller (<100 bp) cfDNA fragments [77, 86] (Fig. 2d). In addition to a proportional ctDNA increase, single-stranded sDNA cfDNA libraries also contain elevated mitochondrial and microbial-derived cfDNA [86] and may therefore offer further options for cfDNA analyses.

### Emerging novel exosome technologies

At present, specific detection and isolation of cancer cell-derived exosomes in the circulation is lacking. It is conceivable that mass spectrometry analyses may further identify cell surface markers, such as the aforementioned GPC1 [50], to improve enrichment of cancer cell-derived exosomes. Together with specific mutations, exosomes may then be used, not only to monitor disease courses, but also to detect early stages of cancers.

However, detection and molecular profiling of exosomes remains technically challenging. Recent approaches for high-throughput quantitative analyses of exosomes employing arrays functionalized with antibodies to enable profiling of exosome surface proteins and proteins present in exosome lysates may greatly facilitate the diagnostic potential of exosomes [87].

### Functional CTC studies and CTC-derived explants

Functional CTC studies are highly challenging because of the low number of CTCs that can be retrieved from patient blood. The development of novel CTC culturing technologies is extremely promising in this regard. One study demonstrated that CTCs from chemotherapy-naïve patients with extensive-stage metastatic small cell lung cancer (SCLC) are tumorigenic in immunocompromised mice [88] (Fig. 2e). Patients’ blood was enriched for CTCs and injected into one or both flanks in mice. CTC-derived explants (CDXs) resulted in samples derived from patients with high CTC numbers (>400 CTCs per 7.5 mL). Histopathology and immunohistochemistry confirmed that CDXs represented clinical SCLC, and detailed analyses of their genomes demonstrated that previously described characteristics of SCLC were maintained [88]. The response of CDXs to therapy closely mirrored overall survival of the corresponding patients [88].

In fact, the generation of cell lines from CTCs is an exciting novel field. Recently, the establishment of CTC lines from patients with colon cancer [89] and breast cancer [36, 90] were reported. In prostate cancer, a 3D organoid system allowed the development of a long-term CTC culture [91]. Perhaps one of the most exciting applications of CTC lines is that CDXs may support selection of targeted therapies and may evolve to instrumental tools for drug development. More detailed analyses of CDXs lines, perhaps as recently demonstrated for patient-derived tumor xenografts [92], are warranted to further investigate the potential of this approach.

## Challenges for liquid biopsy applications and how close are we to the clinic

In particular, a more mature understanding of the biology behind ctDNA, CTCs and exosomes will help us understand if the molecular profiles generated from these sources truly reflect the physiological disease state of the patient and if they can help physicians reliably detect and monitor the disease. In order to confirm this, we must uncover the origin and dynamics of these tumor parts in the circulation and furthermore, determine their biological significance and clinical relevance.

Although the exact mechanisms behind the release and dynamics of cfDNA remain unknown, several hypotheses exist to explain the existence of tumor DNA in the bloodstream. Perhaps the most widely accepted theory is that tumor cells release DNA via apoptosis, necrosis, or cell secretion in the tumor microenvironment [14, 93, 94]. Some cancer cases examined had detectable ctDNA levels but no detectable levels of CTCs [13]. Vice versa, a patient with an excessive number of CTCs of more than 100,000 was described, who, despite of progressive disease, had a low ctDNA allelic frequency in the range of merely 2–3% [26]. While in most patients CTC number and ctDNA levels are mutually correlated [26], such cases illustrate that exceptions exist and that the underlying biology of both CTC and ctDNA release is still poorly understood.

Other basic unknowns regarding liquid biopsy implementation in the clinic revolve around questions of whether or not ctDNA does actually indeed offer a full representation of a patient’s cancer, if all existing metastases contribute to the ctDNA, CTCs, and exosomes found in the bloodstream, or if all tumor cells release an equal amount of ctDNA into the circulation. In order to establish to what extent ctDNA represents metastatic heterogeneity, one study followed a patient with metastatic ER-positive and HER2-positive breast cancer over 3 years [95]. The genomic architecture of the disease was inferred from tumor biopsies and plasma samples and, indeed, mutation levels in the plasma samples suggested that ctDNA may allow real-time sampling of multifocal clonal evolution [95]. Conduction of warm autopsies, i.e., rapid tumor characterization within hours of death, might further help answer these questions more fully, as data derived from the tumor post-mortem could be compared to previously collected ctDNA from the patient [96].

It has furthermore been demonstrated that the percentage of ctDNA within total cfDNA can vary greatly between patients from less than 10% to greater than 50% or, as suggested more recently, can even be detected at fractions of 0.01% [13, 19, 97]. However, despite this high variability in ctDNA levels in different cancer patients, numerous studies have shown that intra-patient levels correlate with both tumor burden and progression of disease [14, 17–20, 27, 29, 98–102], giving evidence for the use of ctDNA levels as a proxy measurement of tumor progression and response to therapy. Accordingly, in colorectal cancer, ctDNA analyses revealed how the tumor genome adapts to a given drug schedule and liquid biopsies may therefore guide clinicians in their decision to re-challenge therapies based on the EGFR blockade [98]. For patients with NSCLC, the Food and Drug Administration approved the implementation of cfDNA in *EGFR* mutation analysis, through a test called the “cobas *EGFR* Mutation Test v2” (Roche), which serves as the first blood-based companion diagnostic to test which patients are potential candidates for the drug Tarceva (erlotinib). In a very recent study [103], this kit was used to confirm that patients treated with first-line EGFR tyrosine kinase inhibitor had acquired the *EGFR* T790M (p.Thr790Met) mutation, which confers resistance to first-generation EGFR tyrosine kinase inhibitors [103]. The authors then showed that NSCLC patients with this T790M mutation who were treated with osimertinib had better response rates and progression-free survival than patients treated with platinum therapy [103]. This is a beautiful example in which an invasive lung tissue biopsy was replaced by a plasma DNA-based blood test, i.e., a liquid biopsy, to identify a group of patients who could benefit from a specific treatment. This will likely propel development of further NGS-based *EGFR* mutation detection assays, which are of particular relevance for the Asian population in which *EGFR* mutation-positive lung cancers occur more frequently than in the Caucasian population [104].

However, before liquid biopsies can serve as viable diagnostic assays, pre-analytical steps, such as the collection of biofluid (e.g., blood, serum, plasma), centrifugation settings, isolation reagents, and storage conditions, must be standardized in order to ensure reproducible processing procedures. Furthermore, analytical steps, such as quantification of cfDNA and subsequent mutational analysis, i.e., the NGS assay and sequencing platform itself, must be validated to simulate clinical settings. In addition, sensitivities and specificities of the applied assays must be robust, reproducible, and have the appropriate internal and external quality controls [72]. Perhaps the most imperative step is the need to evaluate the clinical relevance of ctDNA at various time points depending on the application, such as patient stratification, evaluation of treatment response, efficacy, and resistance, as well as validating this data in large multicenter clinical studies [72]. Furthermore, the clinical performance of cfDNA assays must satisfy the requirements of the respective regulative agencies, such as Clinical Laboratory Improvement Amendments in the USA or the genetic testing practices in European countries. In Europe, efforts to harmonize liquid biopsy testing are supported by CANCER-ID, a European consortium supported by Europe’s Innovative Medicines Initiative, which aims at the establishment of standard protocols for and clinical validation of blood-based biomarkers (www.cancer-id.eu/).

## Conclusions

Cancer is a complex, heterogeneic, and dynamic disease involving multiple gene-environment interactions and affects numerous biological pathways. As such, the development of reliable and robust non-invasive platforms represents a vital step towards the promise of precision medicine. Current work in the liquid biopsy field continues to show great potential utility in the diagnosis and stratification of cancer patients and furthermore exemplifies a surrogate method for monitoring treatment response when compared to the tissue biopsy approach. The ease and frequency made possible by serial liquid biopsy collection offers plenty of advantages compared to standard surgical procedures, especially including the opportunity of more rapid course correction of administering therapies. As technological advances continue and further innovations in liquid biopsy methodology arise in parallel, this approach will hopefully enable methods for pre-diagnostic assessment of cancer risk as well. As our knowledge of the biology behind cfDNA improves, so too will the management of cancer patients as the liquid biopsy method becomes one of clinical reality.

## Abbreviations

CAPP-Seq: cancer personalized profiling by deep sequencing
CDXs: CTC-derived explants
cfDNA: circulating free DNA
CTCs: circulating tumor cells
ctDNA: circulating tumor DNA
EGFR: epidermal growth factor receptor
GPC1: glypican-1
NGS: next-generation sequencing
NSCLC: non-small cell lung cancer
SCLC: small cell lung cancer
SCNAs: somatic copy number alterations
TSS: transcription start site
